# Economic Evaluation alongside Multinational Studies: A Systematic Review of Empirical Studies

**DOI:** 10.1371/journal.pone.0131949

**Published:** 2015-06-29

**Authors:** Raymond Oppong, Sue Jowett, Tracy E. Roberts

**Affiliations:** Health Economics Unit, School of Health and Population Sciences, Public Health Building, University of Birmingham, Birmingham, United Kingdom B15 2TT; Kingston University London, UNITED KINGDOM

## Abstract

**Purpose of the study:**

This study seeks to explore methods for conducting economic evaluations alongside multinational trials by conducting a systematic review of the methods used in practice and the challenges that are typically faced by the researchers who conducted the economic evaluations.

**Methods:**

A review was conducted for the period 2002 to 2012, with potentially relevant articles identified by searching the Medline, Embase and NHS EED databases. Studies were included if they were full economic evaluations conducted alongside a multinational trial.

**Results:**

A total of 44 studies out of a possible 2667 met the inclusion criteria. Methods used for the analyses varied between studies, indicating a lack of consensus on how economic evaluation alongside multinational studies should be carried out. The most common challenge appeared to be related to addressing differences between countries, which potentially hinders the generalisability and transferability of results. Other challenges reported included inadequate sample sizes and choosing cost-effectiveness thresholds.

**Conclusions:**

It is recommended that additional guidelines be developed to aid researchers in this area and that these be based on an understanding of the challenges associated with multinational trials and the strengths and limitations of alternative approaches. Guidelines should focus on ensuring that results will aid decision makers in their individual countries.

## Introduction

Establishing whether new and existing health technologies provide value for money is becoming important internationally, and many countries now require evidence on cost-effectiveness for resource allocation decisions [1–2]. In the UK, the National Institute for Health and Care Excellence appraises health technologies in terms of their clinical effectiveness and cost-effectiveness [3], and economic evaluation, which is the comparison of alternatives in terms of costs and benefits, is one of the tools used for this purpose [4]. Economic evaluations have been conducted alongside trials mainly because they provide a means for collecting clinical and economic data simultaneously. One type of trial that has seen an increase in its popularity, owing to its ability to recruit participants rapidly and expedite the development of new health technologies, is the multinational trial, which is defined as a study that takes place in more than one country or jurisdiction [5–9].

One of the reasons for pooling/aggregating clinical data from cross-country studies is the belief that clinical and biological effects are homogenous across countries/jurisdictions [10]. However, the same cannot be said about economic data, owing to the vast differences in health systems, practice patterns, resource use and unit prices between countries, all of which need to be accounted for when conducting an economic evaluation [11–14]. Recent reviews of the literature revealed wide variation in the way these differences are addressed [15–16], indicating a lack of consensus among researchers. Availability of economic data in some countries also poses potential problems for researchers. A study conducted alongside a neurologic trial in 15 countries reported a dearth of unit cost information in some participating countries despite the efforts that were made to obtain these costs [17]. Generalisability (applying the results of a study to a number of countries without needing to adjust for interpretation) and transferability (adapting the results of a study to other countries) are other challenges that have been identified in the literature [18]. In principle, because of their very nature, results from cross-country studies should be more generalisable. However, it can be argued that pooled results cannot be applied to a single country owing to the inclusion of data from different jurisdictions [19]. A recent review of national guidelines on the use of data from multinational trials showed that there were vast differences in the data different countries considered to be generalisable or transferable to their settings [1], which indicates a lack of consensus among countries and which potentially limits the usefulness of cost-effectiveness estimates from multinational trials [19–20]. Resource allocation decisions are normally made at a national level [20–22], and thus there is the need to develop and agree on appropriate methods for conducting and interpreting economic analyses based on multinational trials. This would not only make results more useful to decision makers but also avoid the duplication of work in every country/jurisdiction [2].

A number of methods have been developed in response to the challenges outlined, ranging from very simple approaches such as adjusting resource use to very complex statistical approaches such as multilevel modelling [22]. The extent to which these methods have been used in practice is unclear. One study concluded that there is a need for more guidance as a result of the vast variation in methods that are being used to conduct economic analyses alongside multinational trials [15]. It is our belief that a study designed to assess the challenges reported by researchers could lead to a better understanding of the reasons methods vary and also help to develop additional guidance in this area. The objective of this study is to review published economic evaluations that were conducted alongside multinational trials with the aim of exploring methods that have been used and to outline researchers’ challenges i.e. any difficulties associated with the multinational nature of the trial. As far as we are aware, no other review has considered the challenges that have been reported by researchers who have conducted economic evaluations alongside multinational trials.

## Materials and Methods

A systematic review was conducted following the guidelines of the Centre for Reviews and Disseminations (CRD) [23].

### Inclusion and exclusion criteria

Studies were included if they were full economic evaluations based on multinational trials and reported an incremental cost-effectiveness ratio (ICER) or incremental net benefit. Studies were excluded if they were modelling studies or systematic reviews, did not use patient-level data or were not published in English.

### Search strategy

The electronic databases searched were: MEDLINE, EMBASE and the National Health Service economic evaluation database (NHS EED). The search was limited to the period 2002 to 2012 for pragmatic reasons and to capture the most recent studies. The following keywords were used in the search: multinational, cost, cost-effectiveness, cost-utility, cost-benefit, multi-country, multi-centre, trial, economic evaluation, and cross-country (S1 Table). Following an approach used by Roberts and colleagues [24], a three-stage process was used to select relevant papers (S1 Text). The screening of papers was done by all reviewers. Stage one (categorization of studies) was carried out by one reviewer who initially screened titles and abstracts of articles and classified them into 5 groups. Stage 2 (further classification of studies) and stage 3 (application of the inclusion criteria) were carried out independently by all three reviewers (see S1 Text). Results from each reviewer were compared and any differences were resolved through consultation among all reviewers. The quality of the economic evaluations was not assessed because of the study objectives and the need to include as many studies as possible.

### Data Extraction

Data were extracted using a predefined data extraction form (S2 Table), and the following data were extracted from the included studies: Type of economic evaluation, health outcomes considered, study perspective, number of countries included, analytical approach to the economic evaluation used and challenges faced.

## Results

**Fig 1:**
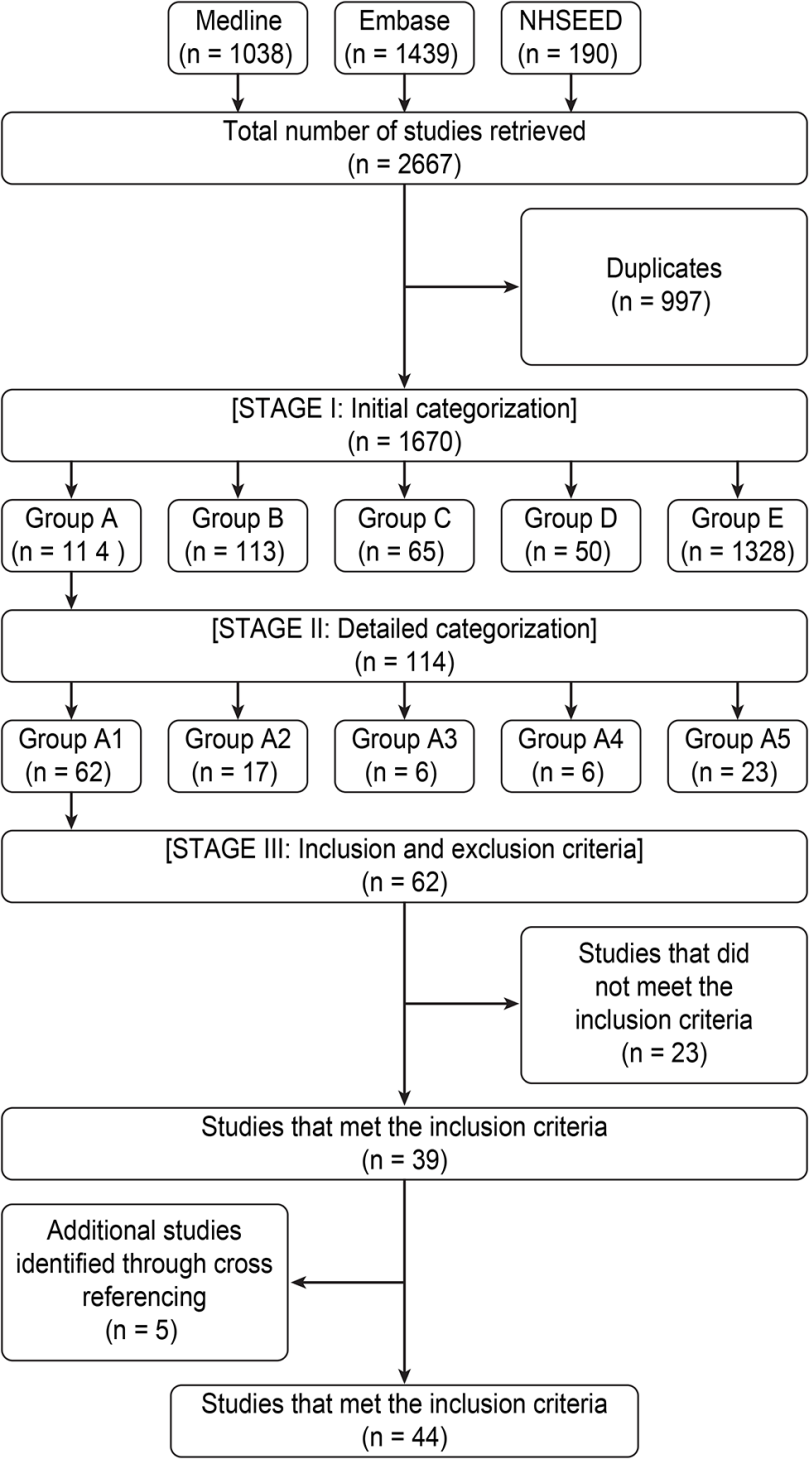
Literature search and selection.

The database searches yielded 2667 articles. After accounting for duplicates, 997 were excluded. Inspecting the titles and abstracts of the remaining papers yielded 114 potentially relevant articles, of which 62 were classified as economic evaluations that reported an ICER or incremental net benefit. Of these, 39 met the inclusion criteria and the remaining 23 were excluded mainly because they were model-based (20 studies) or not relevant (3 studies). An additional 5 studies were identified through cross referencing. Forty-four studies were included in the final sample (Fig 1).

### Summary of selected studies

The types of economic evaluations were mainly cost-effectiveness analysis (31 studies) and cost-utility analysis (18 studies) (Table 1). Of these, 5 studies conducted both [25–29]. In one study, cost-utility analysis was performed as secondary analysis but an ICER was not estimated [25]. Sixteen studies were related to cardiovascular disease, representing a substantial proportion of the included papers. A total of 21 trials were placebo controlled (Table 2), with a common characteristic being their assessment of drug therapies. The number of countries included in an individual trial ranged from 2 to 48, and approximately 80% of studies included in the review recruited patients from the UK (S3 Table). Using World Bank classifications [30], we identified 38 high-income, 24 upper middle-income, 12 lower middle-income and only 5 low-income countries (S3 Table). Only 2 studies included participants from low-income countries: one that assessed interventions for preeclampsia [31] and another that evaluated a malaria intervention [32]. Studies that recruited patients from lower middle-income countries primarily assessed interventions for chronic obstructive pulmonary disorder and asthma [33–36].

**Table 1:** Summary of studies that met the inclusion criteria.

Author/Year	Study aims	Number of countries included (Country EE was carried out)	Type of economic analysis	Health outcomes	EQ-5D Value set used	Study perspective	Analytic approach to the economic evaluation used	Country-specific results presented	Adjustments made to account for country variations	Discussed challenges associated with multinational studies
Canoui-Piotrine et al 2009 [25]	Assess the cost-effectiveness of sirolimus-eluting stents compared with bare metal stents.	15	Cost-effectiveness analysis and cost-utility analysis	Cost per target vessel revascularization avoided	N/A	Health service perspective	Fully split one-country costing	Yes	No	No
Glasziou et al 2010 [26]	Determine the cost-effectiveness of a fixed combination of perindopril and indapamide	20	Cost-effectiveness analysis and cost-utility analysis	Cost per death averted at 4.3 years average follow-up, cost per life year gained and cost per QALY	N/A	Healthcare purchaser perspective	Fully pooled one-country costing	Yes	Yes	Yes
Marcoff et al 2009 [27]	Examine the cost-effectiveness of enoxaparin compared with unfractioned heparin as adjunctive therapy for fibrinolysis	48	Cost-effectiveness analysis and cost utility analysis	Cost per life year gained and cost per QALY gained	N/A	Societal perspective	Fully pooled one-country costing	Yes	Yes Regression approach	Yes
Mittman et al 2009 [28]	Assess the cost-effectiveness of cetuximab in metastatic colorectal cancer	2	Cost-effectiveness and cost-utility analysis	Cost per life year gained and cost per QALY gained	N/A	Payer perspective (Canadian government)	Fully pooled one-country costing	No	No	Yes
Reed et al. 2004 [29]	Estimate the cost-effectiveness of zoledronic acid versus placebo for dressing skeletal complications in men with prostate cancer	17	Cost-effectiveness analysis and cost-utility analysis	Cost per skeletal complication avoided; cost per patient free of skeletal-related event and cost per QALY	N/A	Societal perspective	Fully pooled multi-country costing	No	Yesthrough currency conversion	Yes
Simon et al 2006 [31]	To assess the cost-effectiveness of using magnesium sulfate to prevent preeclampsia	33	Cost-effectiveness analysis	Cost per case of preeclampsia prevented	N/A	Treatment provider perspective (hospital)	Fully pooled multi-country costing	Yes region-/group-specific cost-effectiveness	Yes through currency conversion and country classification	Yes.
Lubell et al 2009 [32]	To explore the cost-effectiveness of artesunate versus quinine for the treatment of severe malaria	4	Cost-effectiveness analysis	Cost per death averted	N/A	Provider perspective	Fully pooled multi-country costing	Yes	Yes	Yes
Sullivan et al. 2003 [33]	Estimate the cost-effectiveness analysis of early intervention with budesonide in mild, persistent asthma	32	Cost-effectiveness analysis	Cost per symptom-free day	N/A	Healthcare payer and societal perspective	Fully pooled one-country costing			Yes
Briggs et al 2006 [34]	Estimate the cost-effectiveness of a single inhaler versus fluticasone proportionate in aiming for total control in asthma patients	44	Cost-utility analysis	Cost per QALY gained	Mapping	Health service perspective	Fully pooled one-country costing	Yes	Yes Regression approach	Yes
Briggs et al 2010 [35]	Inform decision makers about the cost-effectiveness of alternative COPD treatments	42	Cost-utility analysis	Cost per QALY gained	UK tariff	Not clear	Fully split multi-country costing	Yes region-specific	Yes	Yes
Lofdal et al 2005 [36]	Compare the healthcare costs and effects of budesonide/formoterol in a single inhaler with those of budesonide and formoterol monotherapies and placebo in patients with COPD	15	Cost-effectiveness analysis	Cost per avoided exacerbation	N/A	Healthcare payer perspective	Fully pooled one-country costing	No	Yes Followed study protocol rigorously in all countries	No
Bachert et al 2007 [37]	Assess the cost-effectiveness of grass allergen tablet compared with symptomatic medication for preventing seasonal grass pollen-induced rhinoconjunctivitis	7	Cost-utility analysis	Cost per QALY gained	UK tariff	Societal perspective	Fully split multi-country costing	Yes	No	Yes
Canonica et al 2007 [38]	Assess the cost-effectiveness of GRAZAX for preventing grass pollen-induced rhinoconjunctivitis	8	Cost-utility analysis	Cost per QALY gained	UK tariff	Societal perspective	Fully pooled multi-country costing	Yes	No	No
Fernandez et al 2005 [39]	Assess the relative cost-effectiveness of escitalopram compared with venlafaxine in patients with major depressive disorder	8	Cost-utility analysis	Cost per QALY gained	UK tariff	Payer perspective	Fully pooled multi-country costing	No	Yes Regression approach	Yes
Manca et al 2003 [40]	Assess the cost-effectiveness of tension-free vaginal tape compared with open burch colposuspension as a primary treatment for urodynamic stress incontinence	2	Cost-utility analysis	Cost per QALY gained	UK tariff	Health service perspective	Fully pooled one-country costing		No	No
Garry et al. 2004 [41]	Evaluate the cost-effectiveness of laparoscopic, abdominal and vaginal hysterectomy	2	Cost-utility analysis	Cost per QALY gained	UK	UK NHS perspective	Fully pooled one-country costing	Yes		
Nasser et al. 2008 [42]	To assess the cost-effectiveness of GRAZAX in patients with rhinoconjunctivitis and coexisting asthma	8	Cost-utility analysis	Cost per QALY gained	UK tariff	Societal perspective	Fully pooled one-country costing	Yes	No	No
Bracco et al 2007 [43]	Assess the cost-effectiveness of tegaserod in treating irritable bowel syndrome	Not stated	Cost-utility analysis	Cost per QALY gained	Appears to be UK tariff	Third-party payer perspective	Fully pooled one-country costing (check)	No	Yes Regression approach	Yes
Knapp et al 2008 [44]	Determine the cost-utility of treating schizophrenic patients with olanzapine compared with other antipsychotics	10	Cost-utility analysis	Cost per QALY gained	UK tariff	Health service perspective	Fully pooled one-country costing	No	Yes Regression approach	Yes
Buxton et al 2004 [46]	Assess the cost-effectiveness of early intervention with budesonide in mild asthma	32 (Mentioned 8 in paper)	Cost-effectiveness analysis	Cost per symptom free day	N/A	Healthcare payer perspective and societal perspective	Partially split multi-country costing	Yes	Yes Used country-specific costs	Yes
Rutten Von Molken et al 2007 [47]	Assess the cost-effectiveness analysis of roflumilast for treating patients with severe chronic obstructive pulmonary disease	14	Cost-effectiveness analysis	Cost per exacerbation avoided	N/A	Societal and NHS perspectives	Fully pooled one-country costing	No	Yes through currency conversion	Yes
Willan et al 2006 [48]	Assess the cost-effectiveness of rivastigmine in patients with Parkinson’s disease dementia	12	Cost-utility analysis	Cost per QALY gained	N/A	Societal perspective	Fully pooled multi- country costing		Yes Regression approach	Yes
Radeva et al 2005 [49]	Determine the cost-effectiveness of everolimus compared with azathioprine one year after de novo heart transplantation	14	Cost-effectiveness analysis	Cost per additional patient free of efficacy failure	N/A	Societal perspective	Fully pooled multi-country costing	No	Yes Regression approach	No
Edbrooke et al 2011 [50]	To assess the implications of intensive care unit triage decisions on patient mortality	7	Cost-effectiveness analysis	Cost per life-year saved and cost per life year	N/A	Not clear	Fully pooled multi-country costing	No	Yes Regression approach	Yes
Lamy et al 2004 [51]	Assess the cost-effectiveness of the use of clopidogrel in acute coronary syndromes	28	Cost-effectiveness analysis	Cost per CV death prevented	N/A	Societal perspective	Fully pooled multi-country costing	Yes	Yes Regression approach and event costs	Yes
Drummond et al 2003 [52]	Determine the cost-effectiveness of sequential i.v./po moxifloxacin therapy compared with i.v./po co-amoxiclav with or without clarithromycin in treating community-acquired pneumonia	10	Cost-effectiveness analysis	Cost per additional patient cured	N/A	Health service perspective	Fully pooled one country costing	Yes	Yes Regression approach	Yes
Gomes et al. 2010 [53]	Assess the cost-effectiveness of general versus local anesthesia for carotid surgery	24	Cost-effectiveness analysis	Cost per event-free day	N/A	Health service and personal social services	Fully pooled one-country costing	Yes	No	Yes
Lorgelly et al 2010 [55]	Assess the cost-effectiveness of rosuvastatin treatment in systolic heart failure	21	Cost-effectiveness analysis	Cost per major CV event avoided	N/A	Healthcare perspective	Fully pooled one-country costing	No	Yes Used event cost	Yes
Price et al 2002 [57]	Assess the cost-effectiveness of chlorofluorocarbon-free beclomethasone dipropionate in treating chronic asthma	4	Cost-effectiveness analysis	Cost per symptom free day	N/A	Healthcare provider	Fully pooled one-country costing	Yes appeared to be UK	Yes Adjusted resource use	Yes
Weintraub et al 2005 [58]	Assess the long-term cost-effectiveness of clopidogrel in patients with acute coronary syndromes	28	Cost-effectiveness analysis	Cost per life year gained	N/A	Societal perspective	Fully polled one-country costing	Yes	No	Yes
Wade et al 2008 [59]	Evaluate the cost-effectiveness of escitalopram versus duloxetine in treating major depressive disorder	9	Cost-effectiveness analysis	Change in Sheehan Disability Scale	N/A	Societal perspective	Fully pooled one-country costing	No	Yes Regression approach	Yes
Kolm 2007 [60]	Assess the cost-effectiveness of clopidogrel in acute coronary syndromes	28	Cost-effectiveness analysis	Cost per life year gained	N/A	Canadian health system	Fully pooled one-country costing	Yes	Yes	Yes
Jowett et al 2009 [61]	Assess the cost-effectiveness of computer-assisted anticoagulant dosage versus manual dosing in patients on long- or short-term oral anticoagulant therapy	13	Cost-effectiveness analysis	Cost per clinical event avoided	N/A	Healthcare perspective	Fully pooled one-country costing	No	No	Yes
Dukhovny et al 2011 [62]	Evaluate the cost-effectiveness of caffeine for apnea of prematurity	9	Cost-effectiveness analysis	Survival without bronchopulmonary dysplasia (BPD) or neurodevelopmental impairment (NDI)	N/A	Third-party payer perspective	Fully pooled one-country costing	No	Yes Regression approach	Yes
Annemans et al 2003 [81]	Assess the cost-effectiveness of recombinant urate oxidase in hematological cancer patients	4	Cost-effectiveness analysis	Cost per life year saved	N/A	Healthcare payer	Fully pooled multi-country costing	Yes	No	
Aspelin et al 2005 [82]	Assess the cost-effectiveness of iodixanol in patients at high risk of contrast-induced nephropathy	5	Cost-effectiveness analysis	Cost per adverse drug reaction avoided	N/A	Hospital perspective	Fully pooled one-country costing	Yes		No
Bakhai et al. 2003 [83]	Evaluate the cost-effectiveness of coronary stenting and abciximab for patients with acute myocardial infarction	9	Cost-utility analysis	Cost per QALY gained	N/A	Third-party payer perspective	Fully split one-country costing	Yes	No	No
Brown et al. 2003 [84]	Establish the cost-effectiveness of eptifibatide treatment for acute coronary syndrome patients	28	Cost-effectiveness analysis	Cost per life year gained	N/A		Fully split one-country costing	Yes	No	No
Janzon et al 2003 [85]	Assess the cost-effectiveness of extended treatment with low molecular weight heparin (dalteparin) in unstable coronary artery disease	3	Cost-effectiveness analysis	Cost per avoided death or myocardial infarction	N/A	Healthcare provider perspective	Fully pooled one-country costing	No	Yes Tested the impact of price differences between countries	No
Lamy et al 2003 [86]	Assess the cost implication of using ramipril in high-risk patients based on the heart outcomes prevention evaluation (HOPE) study	19	Cost-effectiveness analysis	Cost per primary event saved	N/A	Third-party payer perspective	Fully pooled one-country costing	Yes	No	No
Lindgren et al. 2005 [87]	Assess the cost-effectiveness of formoterol and salbutamol in patients with asthma	24	Cost-effectiveness analysis	Cost per avoided severe exacerbation	N/A	Healthcare payer perspective	Fully pooled multi-country costing	Yes	No	No
Martin et al 2003 [88]	Determine the cost-effectiveness of epoetin-Alfa versus placebo in stage IV breast cancer.	15	Cost-utility analysis	Cost per QALY gained	N/A	Health service perspective	Fully pooled one-country costing (Not clear)	No	No	No
Reed et al 2004 [89]	Assess the cost-effectiveness of valsartan in patients with chronic heart failure	16	Cost-effectiveness analysis	Cost per life year saved	N/A	Societal perspective	Fully pooled multi-country costing	No	Yes Used country-specific costing and other approaches	Yes
Welsch et al 2009 [90]	Cost-effectiveness of enoxaparin compared with unfractionated heparin in ST elevation myocardial infarction patients	48	Cost-effectiveness analysis	Cost per life year gained	NA		Fully pooled one-country costing	Yes	No	Yes

I A fully pooled analysis is a study that relies on resource use and effectiveness data from all participating countries II A fully split analysis is one that relies on resource use and effectiveness from one or a subset of countries. III Partially split analysis relies on effectiveness data from all participating countries but relies on resource use data from one or a subset of countries. IV One-country costing applies the unit cost from one country V Multi-country costing applies unit costs from two or more participating countries.

**Table 2:** Specific characteristics of studies included in the review.

Author	Placebo controlled trial	Provided sources of unit costs in each country	Currency used			
			Pounds	Euro	US dollar	Other
Canoui-Piotrine et al 2009 [25]	✗	✗		✓		
Glasziou et al 2010 [26]	✓	✗				✓
Marcoff et al 2009 [27]	✗	✗			✓	
Mittman et al 2009 [28]	✗	✗			✓	
Reed et al 2004 [29]	✓	✗			✓	
Simon et al 2006 [31]	✓	✗			✓	
Lubell et al 2009 [32]	✗	✗			✓	
Sullivan et al. 2003 [33]	✗	✗			✓	
Briggs et al 2006 [34]	✗	✗	✓			
Briggs et al 2010 [35]	✓	✗			✓	
Lofdal et al 2005 [36]	✓	✗		✓		
Bachert et al 2007 [37]	✓	✓		✓		
Canonica et al 2007 [38]	✓	✓		✓		
Fernandez et al 2005 [39]	✗	✗		✓		
Manca et al 2003 [40]	✗	✗	✓			
Garry et al. 2004 [41]	✗	✗	✓			
Nasser et al. 2008 [42]	✓	✗	✓			
Bracco et al 2007 [43]	✓	✗		✓		
Knapp et al 2008 [44]	✗	✗	✓			
Buxton et al 2004 [46]	✓	✓			✓	
Rutten Von Molken et al 2007 [47]	✓	✗		✓		
Willan et al 2006 [48]	✓	✓	✓			✓
Ra✓deva et al 2005 [49]	✗	✗			✓	
Edbrooke et al 2011 [50]	✗	✗		✓		
Lamy et al 2004 [51]	✓	✗	✓	✓	✓	✓
Drummond et al 2003 [52]	✗	✗		✓		
Gomes et al. 2010 [53]	✗	✗	✓			
Lorgelly et al 2010 [55]	✗	✗	✓			
Price et al 2002 [57]	✗	✗	✓			
Weintraub et al 2005 [58]	✓	✗			✓	
Wade et al 2008 [59]	✗	✗	✓			
Kolm 2007 [60]	✓	✗			✓	
Jowett et al 2009 [61]	✗	✗		✓		
Dukhovny et al 2011 [62]	✓	✗			✓	
Annemans et al 2003 [81]	✗	✗		✓		
Aspelin et al 2005 [82]	✗	✗		✓		
Bakhai et al. 2003 [83]	✗	✗			✓	
Brown et al. 2003 [84]	✗	✗				✓
Janzon et al 2003[85]	✓	✗	✓			✓
Lamy et al 2003 [86]	✓	✗			✓	
Lindgren et al. 2005 [87]	✗	✗		✓		
Martin et al 2003 [88]	✓	✗	✓			
Reed et al. 2004 [89]	✓	✗			✓	
Welsch et al 2009 [90]	✓	✗			✓	

### Health outcomes

Studies that adopted the CEA approach reported general outcomes such as cost per life year gained or used disease-specific outcome measures such as cost per cardiovascular event avoided. The quality adjusted life year (QALY) was the main outcome measure for those that used CUA; however, different methods were used to estimate QALYs. The main approach was to obtain responses to the EQ-5D questionnaire and use them to obtain health utilities (Table 1). Nine studies gave an indication of how they generated EQ-5D index scores [35, 37–44], and in all cases, the UK tariff [45] was used, mainly because it was well established [43], recommended [35] and readily available [44]. Only one study used the Health Utility Index to obtain QALYs [28]. Mapping was another approach used for this purpose; one study used a mapping algorithm to obtain QALYs from the Asthma Quality of Life Questionnaire [34].

### Costing and study perspective

Twenty-eight studies applied unit costs from only one country to the data; the others applied unit costs from all or a subset of countries (Table 1). The average number (range) of countries per study was 17 (2 to 48) and 16 (4 to 42) for studies that adopted the one-country and multi-country approaches, respectively. One reason for adopting a one-country costing approach was the availability of good-quality data in countries such as the UK [44]. Most studies presented results from one perspective (health service/healthcare or societal) (Table 1), although three adopted multiple perspectives for the purpose of comparison [33,46–47]. The results obtained from the different perspectives were comparable [33,47], although one study had results that were sensitive to the perspective adopted [46]. In terms of what was considered societal costs, most studies included productivity losses using human capital [33, 37, 38, 42, 46] or friction costs approaches [47]. One study included caregiver time [48], whereas others were not explicit about what was included.

The level of detail given about the sources of unit costs varied from simply stating that official tariffs and retail prices in each country had been used [37] to providing detailed references of each country’s unit costs [25,46,48]. In most cases, it was unclear how costs had been obtained (Table 2). One approach to costing when unit costs were unavailable was to assume that countries were similar in terms of geographic proximity and level of development and apply the mean cost from countries that were assumed to be similar to the countries for which costs were not available [31,49]. In contrast, the market basket approach, which involves developing an index that reflects the relative costs of a basket of resources used in a pair of countries [17], was used in two studies [29,49]. Other approaches included using recognized international databases such as the WHO-CHOICE database [32], contacting local health economists and researchers through surveys that elicited unit cost information [29,49] and the top-down/macro-costing approach, which considers costs at an aggregate level [50]. This approach has been shown to be effective in cases when obtaining unit costs is not feasible [4]. Some studies used a combination of methods, such as using the market basket approach and contacting local researchers [29, 49]. In terms of presenting costs, the most common currencies used were the US dollar, the Euro and the UK pound, with one study [51] presenting its results using more than one currency (Table 2).

### Analytical approach to economic evaluation

Based on a well-known classification system (S2 Text) [19], 26 studies were classified as fully pooled one-country costing, and 13 were fully pooled multi-country costing studies. Some studies adopted the fully split approach, with 60% of these using one-country costing. One study was classified as a partially split multi-country costing study (S4 Table). The justification for pooling data was that the sample size in some participating countries was too small [37,38], but only one study tested whether it was appropriate to pool data across countries [52].

### Methods for addressing the multinational nature of the data and ensuring the generalisability and transferability of results

#### Estimating country-specific cost-effectiveness

Two studies used subgroup analysis within sensitivity analysis to estimate ICERs using only data from the country of interest [26,53], and in both cases, the results were similar to the main (pooled) analysis. A third study ignored data from all other countries and used data from only the country of interest [25]. Empirical Bayesian shrinkage, a method that involves borrowing strength from the overall trial to estimate country-specific cost-effectiveness [54], was used by only one study; however, the authors did not present the country-specific estimates [27]. The simplest approach was to state that the perspective of the analysis was related to a particular country and to apply unit costs from that country to the trial-wide data [34, 36, 37, 46, 52]. With regard to reporting the country-specific results, one study [51] reported the cost-effectiveness results in the country’s own currency, whereas other studies presented their results in currencies such as US dollars or Euros.

#### Regression methods

Multilevel modelling was used in three studies to account for the clustered nature of the data [27, 49, 50]. Other regression approaches such as controlling for country when estimating outcomes such as the QALY [43], adjusting for length of stay and costs within countries [51] and including interaction terms and country dummy variables [34,39,44,52] were also used. In one of the studies, the authors went further to test whether the country dummy variables were significant [39].

#### Other approaches

Event rather than daily costs were used to eliminate effects such as differences in lengths of stay across countries [51,55]. Close adherence to the study protocol [56] was also used to eliminate differences in practice patterns and resource use in different countries [36]. One study made assumptions about the number of visits per patient to reflect current guidelines and the UK Department of Health’s recommendations for the management of asthma [57].

### Challenges associated with the economic evaluation of multinational trials

Potential challenges were discussed in 29 studies (Table 1), including:

#### Differences between countries

It was noted that there are numerous differences between countries but no accepted guidance on how to account for them [35, 46, 53]. These differences include: differences in resource use, prices, health systems and practice patterns [27–29,31,39,46,47,52,54,58–60]. Estimating country-specific cost-effectiveness was another area in which there is no consensus amongst researchers [35,46]. One study acknowledged this and outlined the advantages and disadvantages of some of the approaches that had been suggested in the literature [35].

#### Sample size and lack of data

Sample size problems were mentioned by some researchers, who noted that uneven recruitment across countries could potentially lead to unreliable cost-effectiveness estimates, especially in cases in which pooling data across all countries is not an option [31, 32, 35, 37, 46]. The lack of country-specific price weights/costs and the challenges associated with collecting data in multi-country studies were also highlighted in some studies [49, 59, 61]. Most often, the researchers conducting the economic analysis were based in one country and were unlikely to know the sources of unit costs in other countries. In addition, there is also a lack of good-quality data in some participating countries, particularly in developing countries [29, 44, 62]. One study was aware of the advantage of using country-specific price weights but went on to use price weights from only one country [62].

#### Additional challenges

The cost-effectiveness threshold, which represents society’s willingness to pay for an additional unit of benefit, is often used to determine whether an intervention is cost-effective [63]. However, with regard to analyzing multinational trials, researchers are faced with the problem of how to determine and choose the appropriate threshold [28, 34, 43, 46]. One study adopted a threshold of €50,000 per QALY but stated that the decision was based on what other studies had done in the past [43]. Another important issue relates to the generalisability of study findings. Two studies noted that owing to the multinational nature of the data, decision makers in various countries might face problems with making judgments about the cost-effectiveness of interventions in their own country/jurisdiction [29, 35]. Finally, only one study mentioned the choice of the EQ-5D tariff as a challenge [44].

## Discussion

### Summary of main findings

This review has assessed published economic evaluations that were conducted alongside multinational trials. The results indicate that most studies applied costs from one country but resource use from all countries, possibly owing to a lack of cost data in some countries or to the fact that researchers sought to inform decisions in a particular country. However, of the studies that reported results from a single country, 50% of them applied one-country costing. The major problem that has been associated with this approach is the possibility of overestimating or underestimating costs [19, 22, 64].

Most studies did not give reasons for having pooled resource use and effectiveness data, although it can be inferred that increasing sample size is a possible motivation for this. One study did test for heterogeneity and homogeneity before pooling data [53]. With regard to pooling resource use, unless the study protocol is followed rigidly, issues related to practice patterns across countries could potentially affect the analysis [19]. However, it should be noted that although protocols have the potential to reduce differences in treatment patterns across countries, they do not necessarily dictate all care provided.

The UK tariff was used in all studies that used the EQ-5D questionnaire to elicit information on health-related quality of life, and although its widespread use can be attributed to its availability [35, 43, 44], it is also possible that other tariffs such as the EU tariff, which was derived from 6 countries, were not used because they are based on the visual analogue scale (VAS). Although some researchers believe that the VAS should not be used in resource allocation decisions because the values obtained are not considered to be utilities [65–67], current research is exploring the predictive value of the EQ-VAS for EQ-5D utilities [68]. In addition to this, most of the studies that used the UK tariff over the EU tariff were published after 2003, the year the EU tariff was published. This supports the findings from other research papers that the UK tariff is most often used [67]. The choice of the EQ-5D tariff is important because different tariffs could lead to conflicting results [69], and the EuroQol group’s current guidance states that the most relevant should be used [70]. However, when the study is multinational, it is difficult to determine the most relevant tariff, and thus, there is a need for further research. A recent study has suggested that researchers explore the potential for different results using all appropriate tariffs within sensitivity analyses [71].

A number of studies made some form of adjustment to the data to account for the multinational nature; however, the methods used varied, indicating that methods have not been standardized in this area. With regard to studies that looked at country-specific results, only one study explicitly stated that the reason for doing this was the important role of health economics in policy making [46]. The most common method of obtaining country-specific estimates was fully pooled one-country costing. Current recommendations by the ISPOR taskforce suggest that the more complex methods such as hierarchical modeling should be used for the analysis of multinational trial data [18] and a recent study also concluded that Bayesian hierarchical models are the most appropriate for estimating country-specific cost-effectiveness [22]; however, only one study in this review used this approach [27], suggesting that researchers are not adhering to existing guidelines, possibly because of the complexity associated with implementing this approach. Bayesian hierarchical models have been challenged because it assumes that differences between countries are random, whereas in reality, these differences are systematic [72].

The multinational nature of the data was acknowledged by most studies, but not all listed the countries that were included, and some merely reported the number of countries in the trial. This may be attributable to word limits imposed by journals. In most cases, it was not clear whether the study was attempting to estimate general or country-specific results, primarily as a result of inadequate reporting. With respect to unit costs, we found that the sources of the costs were not stated in most cases. This is of great concern because this information would enable researchers and decision makers to judge the validity of the study and whether it was applicable to their own settings and also help other researchers identify unit cost sources. It is therefore advisable that future multinational studies include unit cost sources, and if assumptions about the unit costs were made, this should also be made explicit. This review also found that recruitment is biased towards developed countries, which may reflect the difficulties associated with recruiting patients and the lack of high-quality data in low-income countries [44].

#### Comparison with other studies

Other reviews have looked at economic evaluations alongside multinational trials and obtained results similar to what was found in our study. One study found that reporting on economic evaluations of multinational trials is inadequate [16], another found that methods of analysis differed between studies [15] and a recent review reported that the uptake of the more complicated methods for estimating country-specific cost-effectiveness is slow [73].

#### Strengths and limitations of the study

As with any systematic review, there is the possibility that some articles may have been missed. However, we made the best attempt to identify all possible studies by developing the search strategy with advice from an information specialist. The key strength of this study is that it documented the challenges that have been reported by researchers who have conducted economic evaluations of clinical trials, and no other systematic review of multinational trials has done this.

#### Implications for current practice and future research

The most frequently mentioned challenge was the differences between countries, which could possibly affect the generalisability of study findings. Most clinical results from multinational trials are generalisable to the countries that participated in the study. However, results from economic evaluations are not easily generalisable [1] because there are differences in economic circumstances and differences in health systems across various countries. Hence, there is the need to consider these issues when countries are being included in trials. However, the requirements for economic evaluation/analysis are not given prominence when countries are being chosen for inclusion in multinational trials, and country selection is based on factors such as convenience [2,22]. Research is ongoing regarding selecting centres for multi-centre clinical trials [74], but this research needs to be extended to selecting countries in multinational trials as well because the countries included in a study could potentially determine the extent to which the study results are generalisable. In addition, a very important finding is that different methods were used by different studies for costing and addressing differences between countries. This is an indication that guidance similar to that which has been developed for standard economic evaluations needs to be developed. Although it can be argued that data from multinational trials may only serve as inputs into decision models which are used in resource allocation decisions at the national level, there is still the need to develop methods that would ensure that these inputs can be made more generalisable and transferable to individual country contexts when the need arises.

A possible solution to the problem of generalisability and transferability is the use of checklists to ensure that the results meet the required standards [75–78]. However, a possible limitation is the fact that individual items on checklists are sometimes equally weighted [79]. Another suggestion is for researchers to conduct economic evaluations using multiple perspectives. For example, the results of a study that considers both a health service and societal perspective may be useful for decision making in both the UK and the Netherlands.

There is evidence from this study that most researchers are aware of some of the issues surrounding economic evaluation alongside multinational trials, but they did not offer solutions to these challenges in most cases. Researchers should therefore endeavor to document the challenges they face to guide future research. The main challenge we identified was how to address the differences between countries, which could be attributed to a lack of consensus on many aspects such as how to estimate country-specific cost-effectiveness. Future research should therefore focus on reaching a consensus about how to address the challenges associated with multinational trials.

## Conclusion

Despite the difficulties associated with multinational studies, their frequency will increase [80]. It is clear that conducting an economic evaluation in every country/jurisdiction is not feasible or efficient, and decision makers are likely to have to resort in some cases to considering results from other countries/jurisdictions to inform their local decision making despite the obvious limitations. Conducting economic evaluations alongside multinational trials is not trivial, and there should be a conscious effort by all stakeholders to constantly improve methodology in this area. We suggest that additional guidelines be developed to aid in using a consistent approach in this area, and this should be based on understanding the challenges associated with multinational trials and comparing alternative approaches. The guidelines should also be focused on ensuring that results can be useful to decision makers in individual countries.

## Supporting Information

S1 PRISMA Checklist PRISMA checklist. (DOCX)

S1 Table Search terms used (Medline and Embase search). (DOCX)

S2 Table Data extraction form. (DOCX)

S3 Table Country and number of appearances. (DOCX)

S4 Table Summary of study characteristics. (DOCX)

S1 Text Summary of the stages used to categorize the studies. (DOCX)

S2 Text Summary of analytical approaches to economic evaluation of multinational trials. (DOCX)
