# Supplementary material for: Economic Evaluation alongside Multinational Studies: A Systematic Review of Empirical Studies
Source: PLoS One. 2015 Jun 29;10(6):e0131949. doi: 10.1371/journal.pone.0131949 (PMC4488296; doi:10.1371/journal.pone.0131949)
Supplement: S1 Table — (DOCX) [file pone.0131949.s002.docx]

**Table S1: Search terms used (Medline and Embase search)**

| 1 | cost$.mp. |
| --- | --- |
| 2 | cost-utility.mp. |
| 3 | cost-benefit.mp. or cost-benefit analysis/ |
| 4 | cost-effectiveness.mp. |
| 5 | economic evaluation$.mp. |
| 6 | (multinational or multi-national).mp. |
| 7 | (multicentre or multi-centre).mp. |
| 8 | cross country.mp. |
| 9 | trial$.mp. or exp Clinical Trial/ |
| 10 | or/1-5 |
| 11 | or/6-8 |
| 12 | 9 and 10 and 11 |
| 13 | limit 12 to yr="2002 - 2012" |
