# Supplementary material for: Economic Evaluation alongside Multinational Studies: A Systematic Review of Empirical Studies
Source: PLoS One. 2015 Jun 29;10(6):e0131949. doi: 10.1371/journal.pone.0131949 (PMC4488296; doi:10.1371/journal.pone.0131949)
Supplement: S2 Table — (DOCX) [file pone.0131949.s003.docx]

**Table S2: Data extraction form**

| Author/Year |  |
| --- | --- |
| Study aims |  |
| Number of countries included (Country EE was carried out) |  |
| Type of economic analysis |  |
| Health outcomes |  |
| EQ-5D Value set used |  |
| Study perspective |  |
| Analytic approach to economic evaluation used |  |
| Country specific results presented |  |
| Adjustments made to account for variation in country |  |
| Discussed challenges associated with Multinational studies |  |
