# Supplementary material for: Economic Evaluation alongside Multinational Studies: A Systematic Review of Empirical Studies
Source: PLoS One. 2015 Jun 29;10(6):e0131949. doi: 10.1371/journal.pone.0131949 (PMC4488296; doi:10.1371/journal.pone.0131949)
Supplement: S3 Table — (DOCX) [file pone.0131949.s004.docx]

**Table S3: Country and number of appearances**

| **Country** | **no of appearances** | **percentage** | **Income classification (World bank July 2012)** |
| --- | --- | --- | --- |
| UK | 36 | 81.82% | High income |
| Germany | 30 | 68.18% | High income |
| France | 29 | 65.91% | High income |
| Spain | 29 | 65.91% | High income |
| Italy | 26 | 59.09% | High income |
| Netherlands | 24 | 54.55% | High income |
| Belgium | 23 | 52.27% | High income |
| Denmark | 23 | 52.27% | High income |
| Sweden | 22 | 50.00% | High income |
| Australia | 21 | 47.73% | High income |
| Austria | 20 | 45.45% | High income |
| Canada | 20 | 45.45% | High income |
| Poland | 19 | 43.18% | High income |
| Portugal | 18 | 40.91% | High income |
| Hungary | 18 | 40.91% | High income |
| South Africa | 18 | 40.91% | Upper middle income |
| USA | 16 | 36.36% | High income |
| Norway | 15 | 34.09% | High income |
| Switzerland | 14 | 31.82% | High income |
| Finland | 13 | 29.55% | High income |
| Argentina | 13 | 29.55% | Upper middle income |
| Greece | 13 | 29.55% | High income |
| Ireland | 13 | 29.55% | High income |
| Czech Republic | 11 | 25.00% | High income |
| Brazil | 11 | 25.00% | Upper middle income |
| Israel | 11 | 25.00% | High income |
| Mexico | 11 | 25.00% | Upper middle income |
| China | 9 | 20.45% | Upper middle income |
| Malaysia | 9 | 20.45% | Upper middle income |
| New Zealand | 9 | 20.45% | High income |
| Russia | 9 | 20.45% | Upper middle income |
| Thailand | 8 | 18.18% | Upper middle income |
| Slovakia | 7 | 15.91% | High income |
| Chile | 6 | 13.64% | Upper middle income |
| Estonia | 6 | 13.64% | High income |
| Latvia | 6 | 13.64% | Upper middle income |
| Lithuania | 6 | 13.64% | Upper middle income |
| Singapore | 6 | 13.64% | High income |
| Croatia | 5 | 11.36% | High income |
| Romania | 5 | 11.36% | Upper middle income |
| Taiwan | 5 | 11.36% | Upper middle income |
| Turkey | 5 | 11.36% | Upper middle income |
| Bulgaria | 4 | 9.09% | Upper middle income |
| India | 4 | 9.09% | Lower middle income |
| Turkey | 4 | 9.09% | Upper middle income |
| Ukraine | 4 | 9.09% | Lower middle income |
| Belarus | 3 | 6.67% | Upper middle income |
| Hong Kong | 3 | 6.67% | High income |
| Jordan | 3 | 6.82% | Upper middle income |
| Korea | 3 | 6.82% | High income |
| Philippines | 3 | 6.82% | Lower middle income |
| Uruguay | 3 | 6.82% | Upper middle income |
| Iceland | 2 | 4.55% | High income |
| Indonesia | 2 | 4.55% | Lower middle income |
| Lebanon | 2 | 4.55% | Upper middle income |
| Malta | 2 | 4.55% | High income |
| Slovenia | 2 | 4.55% | High income |
| South Korea | 2 | 4.55% | High income |
| Albania | 1 | 2.27% | Lower middle income |
| Bangladesh | 1 | 2.27% | Low income |
| Cuba | 1 | 2.27% | Upper middle income |
| Egypt | 1 | 2.27% | Lower middle income |
| Georgia | 1 | 2.27% | Lower middle income |
| Ghana | 1 | 2.27% | Lower middle income |
| Luxemburg | 1 | 2.27% | High income |
| Malawi | 1 | 2.27% | Low income |
| Nigeria | 1 | 2.27% | Lower middle income |
| Pakistan | 1 | 2.27% | Lower middle income |
| Peru | 1 | 2.27% | Upper middle income |
| Puerto rico | 1 | 2.27% | High income |
| Saudi Arabia | 1 | 2.27% | High income |
| Serbia | 1 | 2.27% | Upper middle income |
| Sierra Leone | 1 | 2.27% | Low income |
| Sri Lanka | 1 | 2.27% | Lower middle income |
| Uganda | 1 | 2.27% | Low income |
| United Arab emirates | 1 | 2.27% | High income |
| Venezuela | 1 | 2.27% | Upper middle income |
| Yemen | 1 | 2.27% | Lower middle income |
| Zimbabwe | 1 | 2.27% | Low income |
