# Supplementary material for: Economic Evaluation alongside Multinational Studies: A Systematic Review of Empirical Studies
Source: PLoS One. 2015 Jun 29;10(6):e0131949. doi: 10.1371/journal.pone.0131949 (PMC4488296; doi:10.1371/journal.pone.0131949)
Supplement: S4 Table — (DOCX) [file pone.0131949.s005.docx]

**Table S4: Summary of study characteristics**

| **Study characteristics** | **Number of studies** | **Percentage** |
| --- | --- | --- |
| Cost-effectiveness analysis | 31 | 70% |
| Cost-utility analysis | 18 | 41% |
| Conducted both cost-effectiveness and cost-utility analysis | 5 | 11% |
| Discussed Challenges | 29 | 66% |
| Fully pooled one country costing | 26 | 59% |
| Fully pooled multi-country costing | 13 | 30% |
| Fully split one country costing | 3 | 7% |
| Fully split multi country costing | 2 | 5% |
| Partially split multi country costing | 1 | 2% |
| Made adjustments to account for multinational nature of study | 25 | 57% |
| Provided sources of unit cost in each country | 4 | 9% |
| Number of studies recruiting in less than 5 countries | 8 | 18% |
| Number of studies recruiting more than 10 countries | 27 | 61% |
