# Supplementary material for: Economic Evaluation alongside Multinational Studies: A Systematic Review of Empirical Studies
Source: PLoS One. 2015 Jun 29;10(6):e0131949. doi: 10.1371/journal.pone.0131949 (PMC4488296; doi:10.1371/journal.pone.0131949)
Supplement: S1 Text — (DOCX) [file pone.0131949.s006.docx]

**Text S1: Summary of the stages used categorize the studies**

***Stage I: Categorization of studies***

The studies identified through the literature search were classified into the following five groups based on an inspection of the titles and abstracts.

1. The study is multinational and includes a full economic evaluation.
2. The study is multinational and reports on costs and/or outcomes but is not a full economic evaluation.
3. The study does not fall clearly into categories (A) or (B) but could have relevant information.
4. The study discusses issues/methodological aspects relating to economic analysis alongside multinational trials.
5. The study is not relevant.

The groups were determined through an initial scoping of the literature. Studies that fell into category A were deemed relevant whilst studies that fell into other categories were excluded.

***Stage II: Detailed categorisation of studies***

All studies that fell into category A were further categorized into sub groups based on a more detailed inspection.

A1. Economic evaluation that reports an Incremental cost effectiveness ratio (ICER) or Incremental net benefit.

A2. Economic evaluation in that a comparison of cost and outcomes of two or more interventions is presented but for which an ICER is not reported.

A3. Methodological study/study protocol

A4. Systematic review

A5. The study is not relevant.

Studies that fell into category A1 were taken forward and all others excluded. The above groups were determined in such a way as to pick out the most relevant studies.

***Stage III: Inclusion and exclusion criteria***

Studies that fell into category A1 were included if the economic evaluation was carried out alongside a randomised or non-randomised multinational clinical trial. Studies were excluded if they were modelling studies or if they did not use patient level data.
