# Supplementary material for: Economic Evaluation alongside Multinational Studies: A Systematic Review of Empirical Studies
Source: PLoS One. 2015 Jun 29;10(6):e0131949. doi: 10.1371/journal.pone.0131949 (PMC4488296; doi:10.1371/journal.pone.0131949)
Supplement: S2 Text — (DOCX) [file pone.0131949.s007.docx]

**Text S2: Summary of analytical approaches to economic evaluation of multinational trials**

**Fully pooled one country costing:** Uses resource use and effectiveness data from all participating countries and costs from just one country

**Fully pooled multi-country costing:** Uses resource use and effectiveness data from all participating countries and costs from all countries

**Partially split one country costing:** Uses effectiveness data from all countries but resource use is obtained from one or a subset of countries in the trial. Costs from one just one country is used

**Partially split multi-country costing:** Uses effectiveness data from all countries but resource use is obtained from one or a subset of countries in the trial. Costs from all countries

**Fully split one country costing:** Uses resource use and effectiveness data from one or a subset of countries in the trial and costs from just one country.

**Fully split multi-country costing:** Uses resource use and effectiveness data from one or a subset of countries in the trial and costs from all countries.
